# Evaluating clinicians’ teaching performance

**DOI:** 10.1007/s40037-015-0215-7

**Published:** 2015-09-23

**Authors:** Benjamin C.M. Boerebach

**Affiliations:** 1 Professional Performance Research Group, Center for Evidence-Based Education, Academic Medical Center, Amsterdam, The Netherlands; 2 Department of Strategy & Information, University of Amsterdam, Spui 21, 1012WX Amsterdam, The Netherlands

**Keywords:** Teaching performance, Evaluation, Feedback, Role modelling, Psychometrics

## Abstract

Evaluations of clinicians’ teaching performance are usually a preliminary, although essential, activity in quality management and improvement activities. This PhD project focused on testing the validity, reliability and impact of a performance evaluation system named the System of Evaluation of Teaching Qualities (SETQ) across specialities and centres in the Netherlands. The results of this project show that the SETQ questionnaires can provide clinicians with valid and reliable performance feedback that can enhance their teaching performance. Also, we tried to investigate the predictive validity of the SETQ. In conclusion, the SETQ appears to be a helpful tool for improving clinicians’ teaching performance.

## Background

Clinician teachers play an essential role in residency training as they are responsible for educating residents to become excellent clinicians and at the same time to safeguard the quality of patient care. Valid and reliable evaluations of the strengths and weaknesses of clinicians’ teaching performance are usually a preliminary, although essential, activity in quality management and improvement activities [1]. Such evaluations can facilitate clinicians in generating a critical appraisal of their own performance in order to explore necessary follow-up actions to maintain or improve performance [2]. Although there is a strong history of evaluating the role of clinicians in teaching, a complete system with feedback and follow-up to improve clinicians’ teaching performance was often not available [2]. With the purpose to fulfil that need, the System for Evaluation of Teaching Qualities (SETQ) was developed by Lombarts et al. [3, 4].

This thesis focused on testing the validity, reliability and impact of the SETQ across specialities and settings. Most importantly, we studied (1) whether the SETQ instruments (questionnaires) can be used to evaluate clinicians’ teaching performance across specialities and centres with satisfactory validity and reliability and (2) whether clinicians’ teaching performance improved after receiving performance feedback. Finally, to get an indication of the predictive (criterion) validity of the SETQ evaluation scores, we studied (3) how clinicians’ teaching performance scores were related to their role modelling.

### The system for evaluation of teaching qualities (SETQ)

**Fig. 1:**
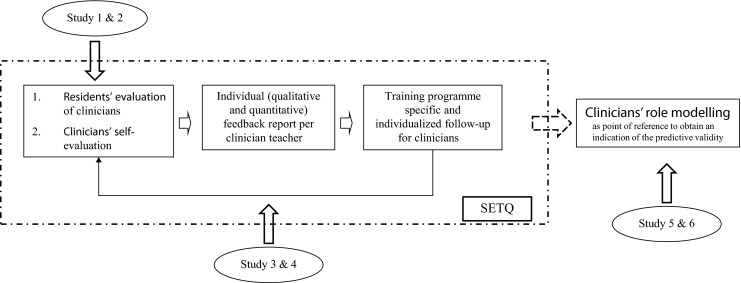
Overview of the SETQ and the studies in this PhD thesis

The SETQ was developed and preliminarily tested by Lombarts et al. (Fig. 1; [3]) In brief, the SETQ uses two questionnaires to gather feedback, one for clinicians’ self-evaluation and one for resident evaluations of clinician teachers. The questionnaires were based on an extensive literature review and discussions with stakeholders [4]. Preliminary analysis by Lombarts et al. [3] showed that the SETQ contained five statistically separate domains of teaching: *learning climate*, *professional attitude towards residents*, *communication of learning goals*, *evaluation of residents*, and *feedback*. In addition to the quantitative data, the SETQ tools request residents to provide narrative comments. Research by Van der Leeuw et al. [5, 6] showed that these narrative comments were appreciated in addition to numerical data because they allow for more specific and detailed feedback. More information about the background and the development of the SETQ can be found elsewhere [3, 4, 6].

## Methods

### Setting and study population

Data were collected at residency training programmes in the Netherlands between September 2008 and October 2013. Collection of the data occurred in phases: an evaluation period at a training programme lasted about a month and was usually repeated annually. Participants were invited to participate by email. The invitation email stressed the formative purpose and use of the evaluations and the confidential and voluntary character of participation. Residents could choose which and how many clinicians to evaluate, based on whose teaching performance the resident felt he or she was able to evaluate accurately. Clinicians could only self-evaluate. After closure of an evaluation period, clinicians received a feedback report, summarizing residents’ feedback along with their self-evaluation. For each study described in this thesis, a purposeful subset of the data was created to sufficiently answer the specific research questions.

### Analyses

In this PhD project, all studies employed quantitative research methods to answer the research questions.

1: Two psychometric validation studies were conducted to study the characteristics of the performance data yielded by both the SETQ resident evaluations and the SETQ self-evaluations. The analysis in study 1 had an exploratory nature and included principal component analysis, reliability analysis and construct validity analysis to study the quality of the SETQ instruments. Study 2 used stronger statistical techniques including a confirmatory factor analysis and generalizability analysis.

2: Two studies aimed to explore the effect of two evaluation cycles which included gathering performance feedback, reporting that feedback to clinicians and individualized follow-up (i.e. the cyclic nature of Fig. 1). The follow-up was not standardized, so it could be adjusted towards the learning goals, experience and preferences of the clinician teachers [7]. It could include a group discussion, guidance by a mentor or additional training (this list is not exhaustive). The follow-up was not studied in this PhD thesis.

Study 3 explored if the teaching performance scores, as evaluated by residents and clinicians themselves, were enhanced after receiving feedback. This study also investigated whether overestimating or underestimating own performance impacted subsequent teaching performance scores. Study 4 explored residents’ perception of clinicians’ teaching performance improvement, after receiving performance feedback for the first and for the second time. One year after clinicians received feedback, residents could rate clinicians’ teaching performance as: not improved, improved, and greatly improved. Besides, we investigated if the number of narrative comments received in a previous evaluation were related to residents ratings of clinicians’ teaching performance improvement in a subsequent evaluation. The positive comments and suggestions for improvement were analyzed separately. Study 3 and 4 used multilevel regression analyses to explore the associations.

3. To obtain an indication of the predictive validity of the SETQ evaluation scores, we related them to clinicians’ role modelling [8]. We choose role modelling as point of reference because it is regarded as an important teaching strategy and no less than 90 % of medical graduates remember role models who shaped their professional skills and attitudes [9, 10]. Clinicians’ role modelling was evaluated by residents through a set of role model items that were separately added to the SETQ questionnaires [8]. We hypothesized that good clinician teachers (with high evaluation scores) would be evaluated as better role models by residents. In study 5, this hypothesis was tested using generalized estimating equation models which related clinicians’ teaching performance scores (overall scores and specific teaching domain scores) to their role modelling. In study 6, we further stress-tested the robustness of our hypothesis, by applying different assumptions of causality and confounding [11].

## Results

1: The results of study 1 and 2 indicated that the SETQ tools appear to be valid for gathering clinicians’ teaching performance data from residents and clinicians’ self-evaluation data. Based on the generalizability analysis of study 2, we can conclude that teaching performance evaluations based on three or more completed SETQ resident evaluations can generate statistically reliable teaching domain and overall scores.

2: The results of study 3 and 4 suggest that clinicians can improve their teaching performance by participating in the teaching performance evaluation system. In study 3 we showed that clinician teachers enhanced their performance scores, after receiving performance feedback for the first and the second time. However, this was not true for clinicians who overestimated their teaching performance during a previous evaluation (compared with the resident evaluations). That subgroup of clinician teachers received lower performance scores during subsequent evaluations. The results of study 4 indicate that residents perceived about 40 % of the clinician teachers to have slightly improved their teaching performance. This was indicated by residents’ ratings in between ‘not improved’ and ‘improved’ (when the ratings given by all residents were aggregated). About 7 % had greatly improved their performance, indicated by aggregated ratings between ‘improved’ and ‘greatly improved’. The strongest predictor of improvement was the number of narrative suggestions for improvement received by clinician teachers during a previous evaluation. The number of positive narrative comments did not predict performance improvement.

3: The results of study 5 showed that relatively strong associations exist between the teaching performance domain scores and clinicians’ role modelling. Overall, displaying a good professional attitude towards residents impacted role modelling most. In study 6, the associations remained—although they differed in strength—under a variety of plausible causal assumptions. The results of these two studies together confirmed our hypothesis that good clinician teachers would be evaluated as better role models by residents and contributed to the predictive validity of the SETQ performance scores.

## Discussion

The SETQ evaluation instruments appear to be valid and reliable in gathering performance feedback from residents. Besides, the SETQ instruments are suitable for both small and larger training programmes where other instruments are more suitable for larger training programmes only [12].

There is a robust body of literature showing that feedback can improve performance [13]. Nevertheless, there is a need for research focussing on *which elements* of feedback are most helpful for improving performance [13]. Our two studies focusing on performance improvement add to this body of knowledge by identifying that narrative suggestions for improvement predicted performance improvement, while narrative positive comments were not associated with performance improvement. Future evaluations should therefore stimulate feedback providers to include suggestions for improvement in their feedback.

Also, we found that overestimating performance resulted in a decline in performance scores. This finding is consistent with findings from the psychological literature which indicate that both overestimating and underestimating performance are frequently identified as constricting factors for performance improvement [14]. Additional research can study whether interventions such as structured guidance in interpreting and appreciating feedback can help overestimating clinicians in benefiting from performance feedback [15]. A recent systematic review found that only a few of the studies on performance evaluation tools assessed the predictive (criterion) validity [16]. In this thesis, we aimed to add to this knowledge by relating teaching performance scores to clinicians’ role modelling. The findings of our studies, which indicate a strong relationship between teaching performance scores and role modelling, strengthen the value of the performance scores. By enhancing their teaching performance, clinicians are likely to also enhance their role modelling, which will make the effect of their teaching more powerful and more effective.

## Conclusions

First, we found that the SETQ can yield valid and reliable evaluations of clinicians’ teaching performance. Second, the performance evaluations can help clinicians in improving their teaching performance. Third, clinicians with higher teaching performance scores were also seen as better role models by residents, and can therefore probably enhance the power of their role modelling by improving their teaching performance.

## Advice for PhD students

Choose your collaborators wisely and carefully. Make sure that enough variety of expertise is grounded in your team. When certain expertise is underrepresented, seek out collaborators outside your current team. You will meet new people, have a lot of interesting interactions, and learn many new useful skills. And above all, it will benefit the quality of your research.

### Funding/Support

This PhD project is part of the research project Quality of Clinical Teachers and Residency Training, which is co-financed by the Dutch Ministry of Health, the Academic Medical Center, Amsterdam, and the Faculty of Health, Medicine and Life Science of the University of Maastricht. Funders had no role in the study designs, data collection, data analysis, data interpretation, or writing of this report.
